# Biochemical Characteristics and Variable Alginate-Degrading Modes of a Novel Bifunctional Endolytic Alginate Lyase

**DOI:** 10.1128/AEM.01608-17

**Published:** 2017-11-16

**Authors:** Yuanyuan Cheng, Dandan Wang, Jingyan Gu, Junge Li, Huihui Liu, Fuchuan Li, Wenjun Han

**Affiliations:** a National Glycoengineering Research Center, Shandong Provincial Key Laboratory of Carbohydrate Chemistry and Glycobiology, and State Key Laboratory of Microbial Technology, Shandong University, Jinan, China; b Department of Food Science and Engineering, Shandong Agriculture and Engineering University, Jinan, China; c Jinan Enlighten Biotechnology Co. Ltd., Jinan, China; Goethe University Frankfurt am Main

**Keywords:** action mode, alginate lyase, Flammeovirga, gene truncation, oligosaccharide-yielding property, substrate degradation pattern

## Abstract

Bifunctional alginate lyases can efficiently degrade alginate comprised of mannuronate (M) and guluronate (G), but their substrate-degrading modes have not been thoroughly elucidated to date. In this study, we present Aly1 as a novel bifunctional endolytic alginate lyase of the genus Flammeovirga. The recombinant enzyme showed optimal activity at 50°C and pH 6.0. The enzyme produced unsaturated disaccharide (UDP2) and trisaccharide fractions as the final main alginate digests. Primary substrate preference tests and further structure identification of various size-defined final oligosaccharide products demonstrated that Aly1 is a bifunctional alginate lyase and prefers G to M. Tetrasaccharide-size fractions are the smallest substrates, and M, G, and UDP2 fractions are the minimal product types. Remarkably, Aly1 can vary its substrate-degrading modes in accordance with the terminus types, molecular sizes, and M/G contents of alginate substrates, producing a series of small size-defined saturated oligosaccharide products from the nonreducing ends of single or different saturated sugar chains and yielding unsaturated products in distinct but restricted patterns. The action mode changes can be partially inhibited by fluorescent labeling at the reducing ends of oligosaccharide substrates. Deletion of the noncatalytic region (NCR) of Aly1 caused weak changes of biochemical characteristics but increased the degradation proportions of small size-defined saturated M-enriched oligosaccharide substrates and unsaturated tetrasaccharide fractions without any size changes of degradable oligosaccharides, thereby enhancing the M preference and enzyme activity. Therefore, our results provided insight into the variable action mode of a novel bifunctional endolytic alginate lyase to inform accurate enzyme use.

**IMPORTANCE** The elucidated endolytic alginate lyases usually degrade substrates into various size-defined unsaturated oligosaccharide products (≥UDP2), and exolytic enzymes yield primarily unsaturated monosaccharide products. However, it is poorly understood whether endolytic enzymes can produce monosaccharide product types when degrading alginate. In this study, we demonstrated that Aly1, a bifunctional alginate lyase of Flammeovirga sp. strain MY04, is endolytic and monosaccharide producing. Using various sugar chains as testing substrates, we also proved that key factors causing Aly1's action mode changes are the terminus types, molecular sizes, and M/G contents of substrates. Furthermore, the NCR fragment's effects on Aly1's biochemical characteristics and alginate-degrading modes and corresponding mechanisms were discovered by gene truncation and enzyme comparison. In summary, this study provides a novel bifunctional endolytic tool and a variable action mode for accurate use in alginate degradation.

## INTRODUCTION

Alginate is one of the most important polysaccharide components of brown seaweeds, e.g., kelp and sargassum (1, 2). Alginate is comprised of α-l-mannuronate (M) and its C_5_ epimer β-d-guluronate (G) units (3). The sugar residues are linked by 1,4-O-glycosides and arranged into linear G-enriched blocks, M-enriched blocks, and the heteropolymer types of MG and GM blocks in the linear molecules. Algal alginate has been widely used as a supporting material in food, medical, and industrial applications for its excellent gel-forming capability after absorbing water (4, 5). Derivatives of M-enriched oligosaccharide fractions have shown potential in treating Alzheimer's disease (6, 7). Recently, the process of enzymatic degradation and bioconversion of algal alginate into alcohol has attracted attention as a solution to non-food-competing energy problems (8, 9). Certain bacteria, including Pseudomonas and Azotobacter strains, can also secrete alginates that contain acetyl modification at the O-2 or O-3 position (10, 11). However, extracellular bacterial alginate has been identified as a macromolecular component of biofilm and a drug barrier in therapy of infections caused by Pseudomonas bacteria (12). Therefore, understanding how to degrade alginate enzymatically and efficiently is an important energetic and medical problem (13).

Alginate lyases can cleave the glycoside linkages in alginate via a β-elimination mechanism (14, 15). Endolytic alginate lyases can degrade alginate into a series of size-defined oligosaccharide fractions, which contain C-4=C-5 double bonds at newly formed nonreducing (NR) ends (16–19). The unsaturated sugar unit is usually designated Δ to show the difference from G and M units. Exolytic alginate lyases can digest alginate by continuously producing monosaccharide products, i.e., G, M, and mainly Δ, from sugar chains (20–22). Moreover, based on substrate preference, alginate lyases are commonly assigned to G-specific, M-specific, and bifunctional groups. Exploration of bifunctional alginate lyases has focused on the enzymes' broad substrate spectrum and excellent alginate degradation efficiency (15, 23). However, relatively little is known about their substrate-degrading modes with regard to the molecular sizes of degradable substrates, the degradation patterns toward different oligosaccharide substrate types, and the structural properties of the final oligosaccharide products, which has limited tool exploration and accurate enzyme use. Moreover, we are interested in whether and why bifunctional alginate lyases are more efficient than substrate-limited enzyme groups in alginate degradation, which is still poorly understood.

Alginate lyases are usually module-organized proteins, comprised of catalytic modules or noncatalytic regions (NCRs) (23). Based on sequence information for their catalytic modules, although a small number of alginate lyases are unclassified, most are assigned to the polysaccharide lyase (PL) families PL5, PL6, PL7, PL14, PL15, PL17, and PL18. Recent studies have investigated the three-dimensional structural characteristics of alginate lyases, including members of the newly defined PL6 family, and have discovered the roles of function modules, conservative motifs, and active-site residues in alginate degradation (24–26). The catalytic modules play essential roles in recognizing, binding, and degrading alginate and thus in determining the enzymes' biochemical characteristics. The NCR fragments cannot degrade alginate but have various effects on the enzymatic properties. For instance, the NCR fragment can assist the catalytic modules of endolytic alginate lyases, e.g., the guluronate lyase Aly5 of Flammeovirga sp. strain MY04 (27) and the bifunctional enzyme Aly2 of Agarivorans sp. strain L11 (28), in binding and degrading small size-defined oligosaccharide substrates efficiently. Interestingly, deletion of the NCR fragment of Aly2 markedly enhanced the M-degrading efficiency of the truncated protein, i.e., changed the substrate preference (28), whereas similar protein truncation of Aly5 caused few changes in substrate preference (27). Therefore, we are interested in why and how the NCR truncation varies the substrate preference of a bifunctional alginate lyase but not that of a substrate-limited lyase.

Bacterial strains within the Flammeovirga genus can efficiently digest and utilize multiple polysaccharides, e.g., agarose, alginate, and starch (29–32). Therefore, they are potential resources of diverse polysaccharide-degrading enzymes. Although many endolytic (33–36) and exolytic (37) agarases have been identified in the genus Flammeovirga, only one G-specific endolytic alginate lyase, Aly5, has been elucidated (27). In this study, we present the protein Aly1 as a novel alginate lyase of Flammeovirga bacteria. In addition, the biochemical characteristics, oligosaccharide-yielding properties, and corresponding substrate-degrading patterns of Aly1 were compared with those of the NCR-truncated protein.

## RESULTS

### Aly1 gene and protein sequence information.

In the genome of Flammeovirga sp. strain MY04, ORF2539 was predicted to encode a candidate polysaccharide lyase (GenBank accession no. ANQ49908). The gene was 1,335 bp with a GC content of 38.5%. The putative protein Aly1 comprises 444 amino acid residues with an apparent molecular mass of ∼50.65 kDa. The predicted isoelectric point (pI) was 4.24.

**FIG 1:**
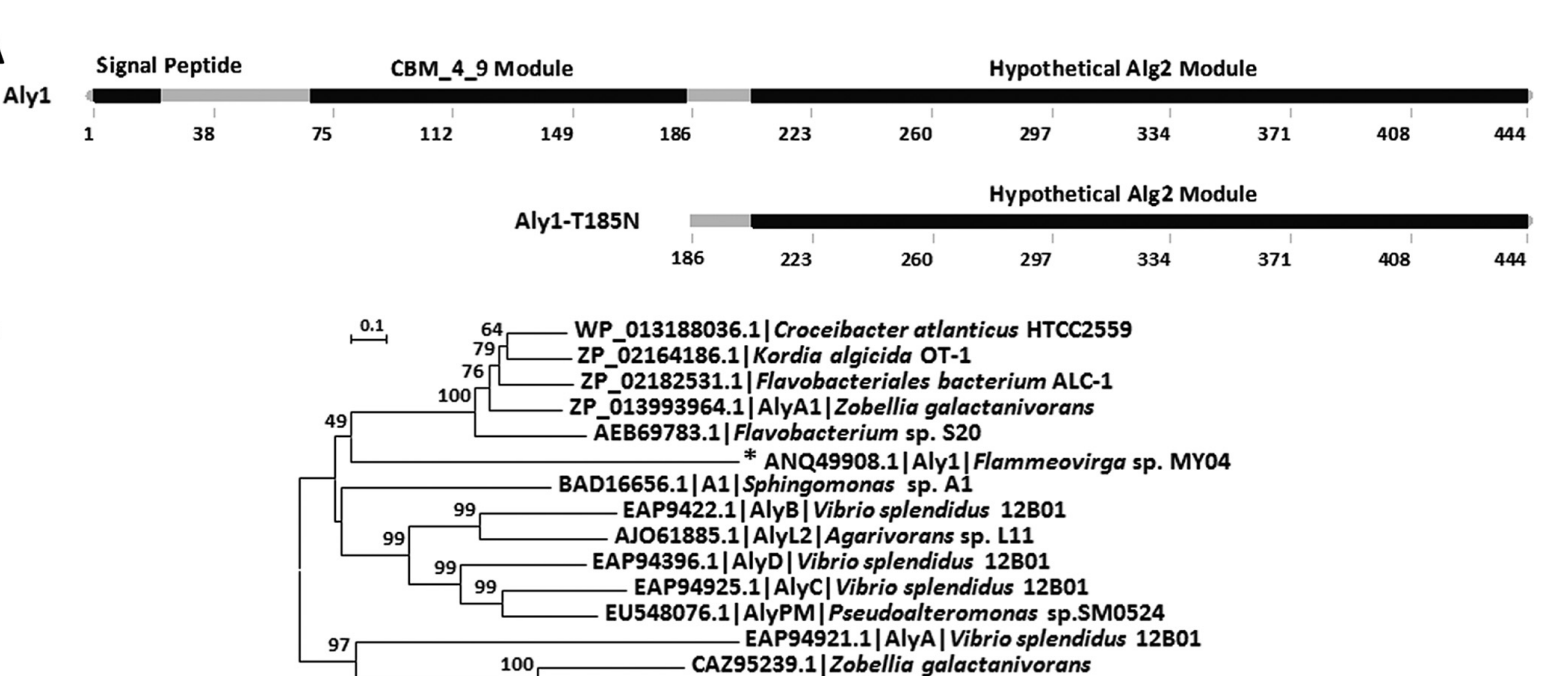
Sequence properties of bifunctional endolytic alginate lyase Aly1 from Flammeovirga sp. strain MY04 and truncated protein Aly1-T185N. (A) Module organizations of Aly1 and Aly1-T185N. The putative N-terminal signal peptide contains 21 amino acid residues. The CBM_4_9 module is a predicted carbohydrate-binding module (Ser^68^ to Glu^184^), and the hypothetical C-terminal Alg2 module (Leu^204^ to Tyr^444^) is a predicted catalytic module of PL7 alginate lyases. (B) Phylogenic analysis of Aly1 and elucidated PL-7 alginate lyases. The tree was created using the neighbor-joining method and MEGA version 6.01 software. The numbers on the branches indicate bootstrap confidence values from 1,000 replicates. The bar is equal to the distance corresponding to 1 amino acid substitution per 10 amino acids.

SignalP 4.1 analysis indicated that the signal peptide of Aly1 contained 21 amino acid residues (Met^1^ to Thr^21^) (Fig. 1A). Analyses using the Carbohydrate-Active Enzyme database and the Simple Modular Architecture Research Tool indicated that the Aly1 protein contains an N-terminal NCR fragment (Asn^22^ to Ser^204^) including a hypothetical carbohydrate-binding module (CBM_4_9) (Ser^68^ to Glu^184^), as well as a C-terminal putative catalytic module, Alg2 (Leu^204^ to Tyr^444^) (Fig. 1A). BLASTp searches showed that both the whole Aly1 protein and its catalytic module Alg2 share sequence identities of below 30% with the elucidated enzymes. Protein sequence alignment showed that in the catalytic module Alg2 the protein Aly1 contains one catalytic motif (Gln^318^-Ile^319^-His^329^ [QIH]), which is conserved in diverse polysaccharide lyases (see Fig. S1 in the supplemental material). Further phylogenetic analysis (Fig. 1B) indicated that Aly1 is a novel member of the PL7 superfamily.

### Protein production and purification of rAly1 and rAly1-T185N.

**FIG 2:**
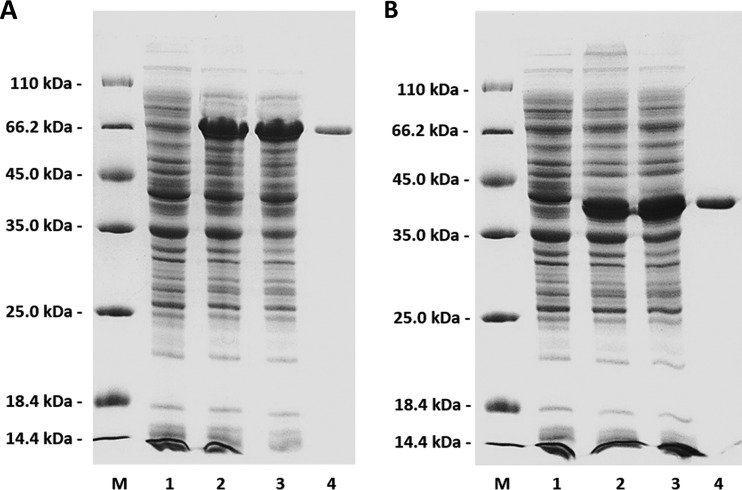
Purification of recombinant Aly1 (A) and NCR-truncated protein rAly1-T185N (B) from E. coli strain BL21(DE3) using Ni^2+^ chelation affinity chromatography. Enzyme purity following each fractionation step was assessed by SDS-PAGE using 13.2% polyacrylamide gels, followed by staining with Coomassie brilliant blue. Lanes M, unstained protein molecular mass marker SM0431 (panels A and B were derived from the same original SDS-PAGE photo and thus share the same molecular mass line); lanes 1, induced cell lysate of E. coli cells harboring control plasmid pET-30a(+); lanes 2, induced cell lysate of E. coli cells containing plasmid pET30-Aly1 (A) or pET30-Aly1-T185N (B); lanes 3, supernatant fluid of the induced cell lysate; lanes 4, rAly1 (A) or rAly1-T185N (B) protein purified from supernatant.

The full-length Aly1 gene was amplified directly from the genomic DNA of Flammeovirga sp. strain MY04. The 1.3-kb PCR products were gel recovered and enzyme cloned into the pET-30a(+) vector downstream of a T7 promoter. A His_6_ tag was successfully added to the C terminus of the protein product (recombinant Aly1 [rAly1]) in the expression vector (pET30-Aly1). To obtain the expression vector of the NCR-truncated protein (rAly1-T185N), the pET30-Aly1 plasmid was amplified using the T185N-F and T185N-R primers. The 6.4-kb PCR products were gel recovered, terminus phosphorylated, and circle ligated to yield the plasmid pET30-Aly1-T185N. SDS-PAGE analyses indicated that BL21(DE3) cells harboring one of the recombinant plasmids (pET30-Aly1 or pET30-Aly1-T185N) could each produce soluble proteins (Fig. 2A and B) with the correct apparent molecular masses and yields of >1.0 g/liter. Using sonication and centrifugation, crude enzymes were each extracted from the Escherichia coli cultures. The protein fractions of rAly1 and rAly1-T185N could be eluted from an Ni-nitrilotriacetic acid (NTA) column using imidazole at concentrations above 50 mM. SDS-PAGE analyses indicated that the purified soluble rAly1 and rAly1-T185N proteins each had purities of greater than 99% (Fig. 2A and B) and initial concentrations of ∼10 mg/ml. Interestingly, native PAGE analyses showed that the two proteins each could form dimers (see Fig. S2 in the supplemental material).

### Enzymatic characteristics of rAly1 and rAly1-T185N.

The two recombinant proteins did not digest chondroitin, chondroitin sulfate, dermantant sulfate B, hyaluronan, heparin, or heparin sulfate but could efficiently digest alginate, M blocks, or G blocks to produce oligosaccharides, exhibiting strong absorbance at 235 nm. The results suggested that Aly1 is a bifunctional alginate lyase. Furthermore, the enzyme activity tests of rAly1 (Table 1) indicated a substrate preference for G over M. The NCR-truncated enzyme of rAly1 showed a substrate preference similar to that of rAly1 (Table 1) and had greater enzyme activities than rAly1 for alginate, G block, and M block substrates.

**TABLE 1:** Enzyme activity analysis for rAly1 and NCR-truncated protein rAly1-T185N

Test substrate	rAly1 activity, U/mg (mean ± SD)	rAly1-T185N	
		Activity, U/mg (mean ± SD)	Relative activity^*a*^
Alginate	1,261 ± 8.2	2,895 ± 4.3	1.34
G block	1,162 ± 3.4	2,259 ± 7.8	1.05
M block	122 ± 4.5	595 ± 7.2	0.28

a Fold activity relative to rAly1’s alginate-degrading activity at an equimolar amount of enzyme.

**FIG 3:**
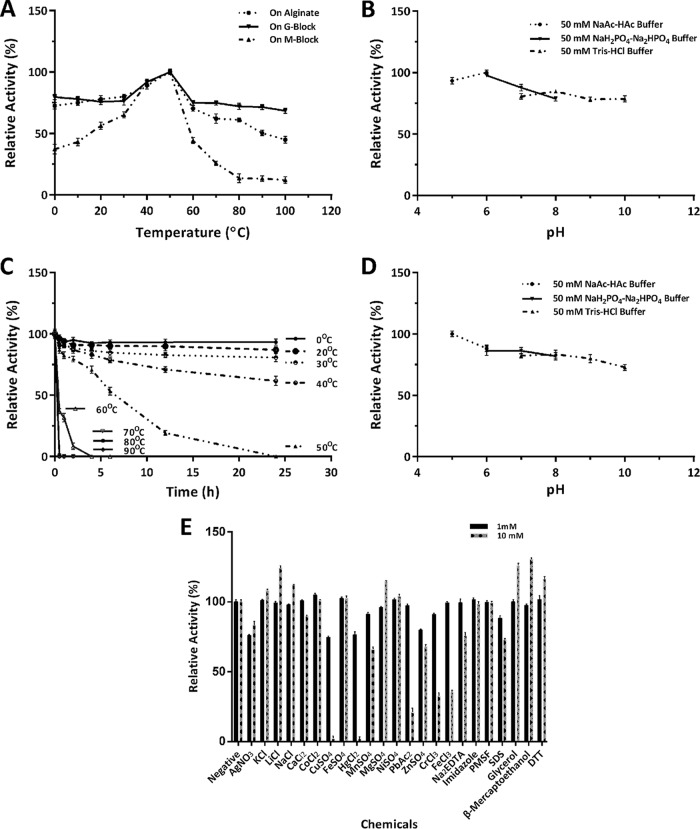
Biochemical characteristics of recombinant bifunctional alginate lyase rAly1. (A) Effects of temperature on enzyme activity toward alginate, G block, and M block. (B) Effects of pH on enzyme activity toward alginate. (C) Thermostability of rAly1 toward alginate. (D) pH stability of rAly1 toward alginate. (E) Effects of chemicals on enzyme activity toward alginate.

The enzyme rAly1 demonstrated the highest activity at 50°C when alginate, M-enriched blocks, or G-enriched blocks were used as substrates (Fig. 3A). A thermostability assay further showed that the alginate-degrading activity of rAly1 was stable at temperatures from 0 to 40°C, and more than 60% activity was retained even if the enzyme was preincubated at the corresponding temperature for 24 h (Fig. 3C). The optimal pH, determined at 50°C in 50 mM sodium acetate-acetic acid (NaAc-HAc) buffer or in 50 mM NaH_2_PO_4_-Na_2_HPO_4_ buffer, was 6.0 (Fig. 3B). The enzyme retained more than 80% of the highest activity after incubation for 2 h in environments ranging from pH 5 to 8 (Fig. 3D). Similar to the case for rAly1, the NCR-truncated enzyme rAly1-T185N showed optimal activity at 40°C and pH 6 (see Fig. S3A and B in the supplemental material). It was also stable at temperatures ranging from 0 to 40°C but retained more than 50% activity after incubation for 24 h (Fig. S3C). Moreover, the truncated enzyme was stable in environments ranging from pH 5 to 7 after incubation at 0°C for 2 h (Fig. S3D).

The alginate lyase activities of rAly1 and rAly1-T185N were strongly inhibited by 10 mM Cu^2+^, Hg^2+^, Pb^2+^, Cr^3+^, or Fe^3+^. In contrast, the enzyme activity of rAly1 was increased to 120 to 130% by 10 mM concentrations of Li^+^, glycerol, and the reducing agent β-mercaptoethanol (Fig. 3E), whereas the activity of rAly1-T185N was increased to 110 to 120% by 1 mM Mg^2+^, and 10 mM Fe^2+^, Mn^2+^, and β-mercaptoethanol (Fig. S3E). Moreover, the activities of the two enzymes were weakly affected by increasing the NaCl concentration from 0 to 1 M.

Under optimal conditions (50°C in 50 mM NaAc-HAc buffer, pH 6.0), rAly1 showed a specific activity of 1,261 ± 8.2 U/mg in alginate degradation, whereas rAly1-T185N exhibited an activity of 2,895 ± 10.3 U/mg, almost 1.3-fold that of rAly1 for an equimolar amount (Table 1).

The results demonstrated that the catalytic module of Aly1 is the key element determining the biochemical characteristics and indicate a bifunctional role in alginate degradation.

### Alginate degradation patterns and oligosaccharide-yielding properties of rAly1 and rAly1-T185N.

**FIG 4:**
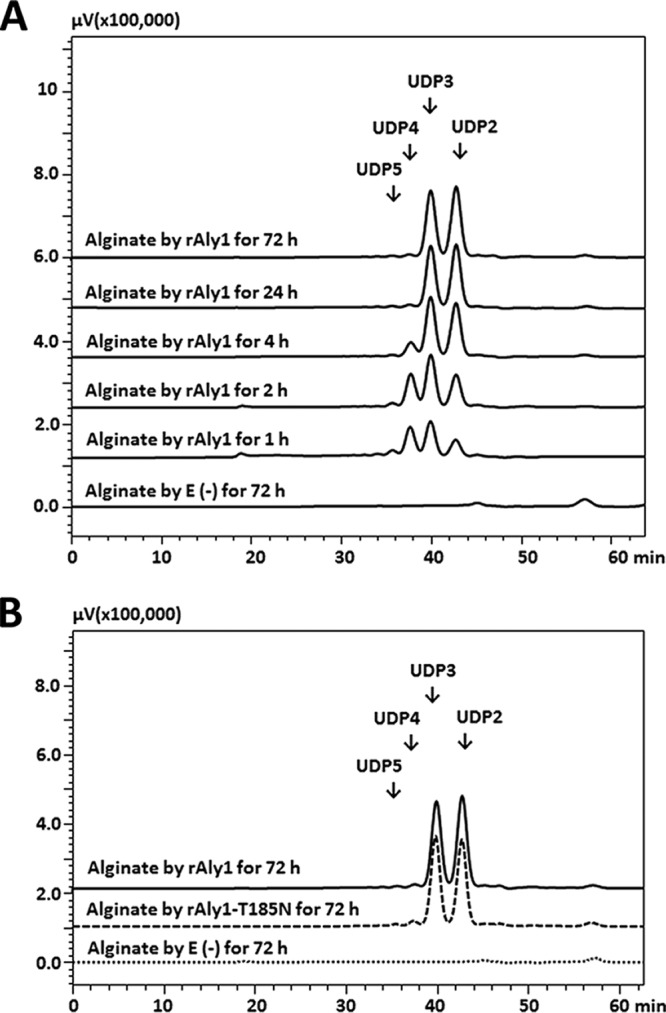
Gel filtration HPLC analyses of alginate digests produced by rAly1 and its NCR-truncated protein rAly1-T185N. (A) Time course analysis of oligosaccharide products of alginate (∼20 μg) reacted with rAly1 (0.1 U/ml) at 50°C. (B) Comparison of final alginate digests produced by rAly1 and rAly1-T185N. E(-), without the enzyme. HPLC analyses were performed using a Superdex peptide 10/300 GL column monitored at a wavelength of 235 nm. UDP2, UDP3, UDP4, and UDP5 represent unsaturated disaccharide, trisaccharide, tetrasaccharide, and pentasaccharide fractions, respectively.

To determine the polysaccharide degradation patterns, alginate (1 mg/ml) was digested by each enzyme (0.1 U/ml) at the corresponding optimal temperatures (rAly1, 50°C; rAly1-T185N, 40°C). The reaction time varied from 0 to 1, 2, 4, 24, or 72 h. Each enzymatic product (∼20 μg) was gel filtered through a Superdex peptide 10/300 GL column and monitored at 235 nm. Similarly, the two enzymes initially produced unsaturated oligosaccharide fractions with high molecular weights and converted them into smaller oligomers and then into a series of oligosaccharides that contained two fractions as the final main products (Fig. 4A and B). The results suggested that the enzyme rAly1-T185N, as well as rAly1, degrades the alginate polysaccharide using an endolytic pattern.

To identify the final oligosaccharide products, a total of 100 mg alginate was digested using each enzyme at optimal temperatures for 72 h. The resulting unsaturated oligosaccharide products were purified into size-defined fractions and analyzed using gel filtration high-pressure liquid chromatography (HPLC). Further mass spectrometry (MS) analyses indicated that the molecular masses of the four smallest oligosaccharide fractions in the final products were 352, 528, 704, and 880 Da. The results indicated that the final alginate digests of each enzyme are comprised mainly of unsaturated disaccharide (UDP2), trisaccharide (UDP3), tetrasaccharide (UDP4), and pentasaccharide (UDP5) fractions. Peak-area analyses demonstrated that the molar ratio of these final products (UDP2/UDP3/UDP4/UDP5) produced by rAly1 was ∼58.5:57.5:2.2:1 (Fig. 4A). Thus, the UDP2 and UDP3 fractions, the final main alginate digests of rAly1, have mass concentrations (wt/wt) of ∼38.2% and ∼57.2%, respectively. The molar ratio of the final products produced by rAly1-T185N (UDP2/UDP3/UDP4) was determined to be ∼50.4:51.5:2.7:1 (Fig. 4B), demonstrating that the UDP2 and UDP3 fractions have mass concentrations (wt/wt) of ∼36.9% and ∼57.3%, respectively. The molar ratio of the final alginate degradation products of each enzyme was the same when the enzyme amount or reaction time were doubled.

**FIG 5:**
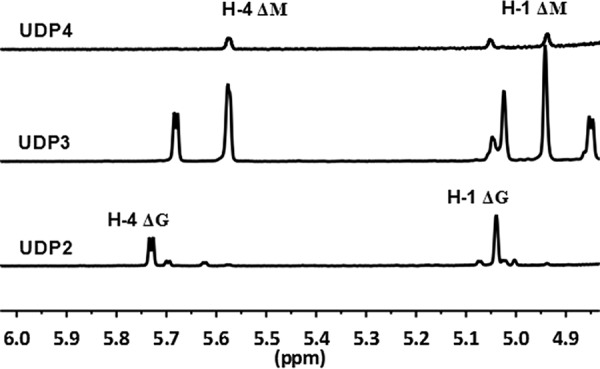
^1^H-NMR analysis (600 MHz, 28°C) of size-defined final main oligosaccharide product fractions produced by rAly1. Final alginate digests were gel filtered using a Superdex peptide 10/300 GL column. Fractions with the same molecular mass (retention time) were monitored at 235 nm and collected for NMR analysis. The H-4Δ signals at 5.70 and 5.74 ppm indicate that the residue neighboring the unsaturated residue is a G, meaning that ΔG constitutes the first two sugar residues at the NR ends. The H-4Δ signals at 5.54 and 5.57 ppm indicate that ΔM constitutes the first two residues at the NR ends. As shown for the UDP3 product fractions, the intensity of the H-4ΔM signals is much higher than that of H-4ΔG signals.

Each purified size-defined oligosaccharide fraction was analyzed by ^1^H nuclear magnetic resonance (^1^H-NMR) spectroscopy to investigate the oligosaccharide-yielding properties of the two enzymes. The UDP2 fractions produced by rAly1 showed strong H-4ΔG signals at 5.75 ppm but weak H-4ΔM signals (Fig. 5) (19). The results indicated that Aly1 produced primarily ΔG units as the UDP2 products in alginate degradation. The UDP3 fractions yielded by rAly1 showed strong signals for H-4ΔG and H-4ΔM at 5.72 and 5.62 ppm, respectively (Fig. 5). Using signal integration, the molar ratio of ΔG to ΔM units within the UDP3 fractions was determined to be ∼67:100. The results indicated that the final main products of UDP3 fractions contain ΔG ends at lower proportions than ΔM ends. The UDP4 fractions showed strong H-4ΔM signals at 5.62 ppm but very weak H-4ΔG signals (Fig. 5). The results suggested that the final oligosaccharide product fractions larger than tetrasaccharides contain only ΔM ends. Therefore, although Aly1 yielded large products containing ΔM units at the NR ends, it preferred to produce small oligosaccharide fractions containing ΔG units, which is a novel oligosaccharide-yielding property reported for bifunctional alginate lyases. The results also demonstrated that similar to the case for Aly5, a G-specific alginate lyase from the same polysaccharide-degrading marine bacterium, Flammeovirga sp. strain MY04 (27), the bifunctional alginate lyase Aly1 exhibits a substrate preference of G to M.

Gel filtration purification by HPLC and further ^1^H-NMR spectrum analyses (see Fig. S4 in the supplemental material) indicated that the NCR-truncated protein rAly1-T185N has an oligosaccharide yield similar to that for the enzyme rAly1, as well as to that for Aly5 of Flammeovirga sp. strain MY04 (27). Therefore, the catalytic module of Aly1 is the key protein element determining the enzyme's G preference and the oligosaccharide products' structural properties in alginate degradation.

### Degradation pattern for unsaturated oligosaccharides prepared using rAly5.

**FIG 6:**
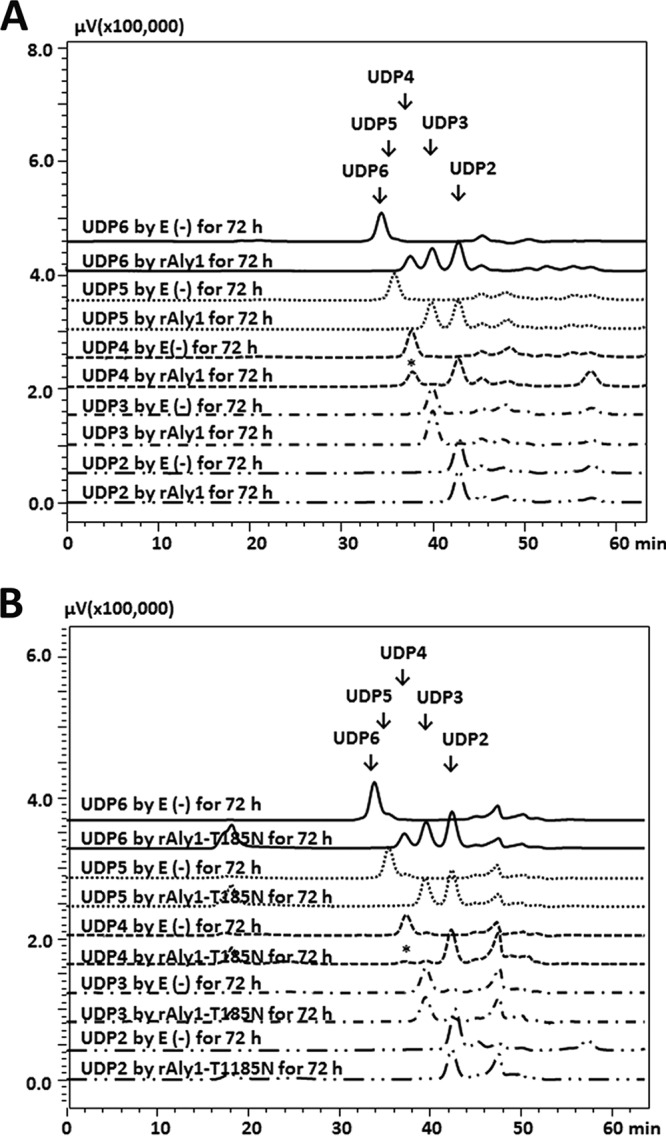
Gel filtration HPLC analyses of final digests of unsaturated oligosaccharide fractions produced by rAly1 (A) and NCR-truncated protein rAly1-T185N (B). Oligosaccharide substrates (∼20 μg) were initially reacted with 0.1 U/ml of rAly1 at 50°C or rAly1-T185N at 40°C. E(-), without the enzyme. An asterisk indicates the enzyme rAly1-T185N degraded UDP4 fractions in proportions greater than rAly1.

To investigate the degradation patterns of the enzymes against associated oligosaccharide substrates, each size-defined unsaturated oligosaccharide chain, i.e., the UDP2, UDP3, UDP4, UDP5, and UDP6 fractions, was reacted with rAly1 and rAly1-T185N using the same strategy as described previously for rAly5 (27). After further enzymatic reaction with rAly1, the final digests of each size-defined oligosaccharide fraction were analyzed via gel filtration HPLC. The results showed that rAly1 could not degrade small size-defined unsaturated oligosaccharide fractions, i.e., the UDP2 and UDP3 fractions (Fig. 6A) but that it could partially digest the UDP4 fraction and could completely degrade larger oligosaccharides, e.g., the UDP5 and UDP6 fractions (Fig. 6A). The enzyme rAly1 could partially process the UDP4 fraction, at a molar proportion of ∼45.7%, into two UDP2 products (Fig. 6A). It could degrade the UDP5 fraction to yield UDP2 and UDP3 fractions in approximately equal molar proportions (Fig. 6A). The enzyme could digest the UDP6 fraction to produce two molecules of UDP3 or two oligosaccharide fractions of UDP2 and UDP4, the latter of which could be further processed into two UDP2 products (Fig. 6A). Therefore, the UDP4 fraction is the smallest unsaturated oligosaccharide substrate, and the UDP2 fraction is the minimal unsaturated oligosaccharide product of rAly1.

When reacted with each size-defined unsaturated oligosaccharide fraction, the NCR-truncated enzyme rAly1-T185N exhibited endolytic patterns similar to those of rAly1, but it could degrade tested UDP4 fractions at a high proportion (∼ 85%) to produce more UDP2 products (Fig. 6B). Thus, the alginate-degrading efficiency of rAly1-T185N is improved by NCR truncation.

### Degradation pattern for saturated oligosaccharides.

**FIG 7:**
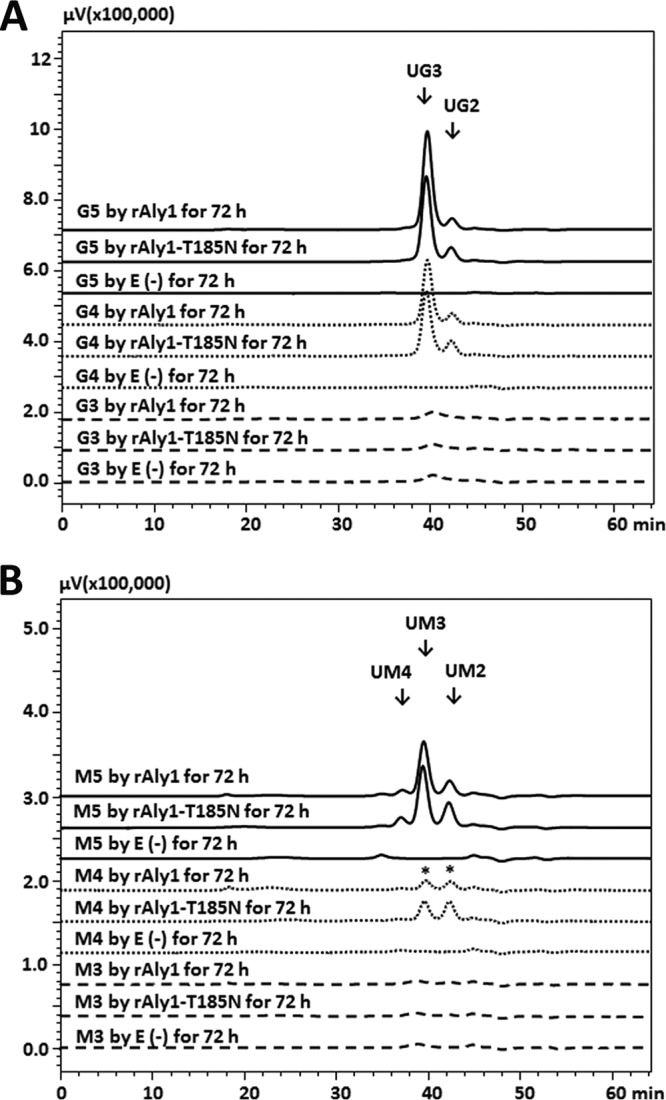
Gel filtration HPLC analyses of final digests of saturated oligosaccharide fractions enriched with G (A) or M (B) produced by rAly1 and NCR-truncated protein rAly1-T185N. E(-), without the enzyme. An asterisk indicates that under the same operation conditions (0.1 U/ml, 40°C), the enzyme rAly1-T185N degraded saturated tetrasaccharide fractions enriched with M (M4) to produce M2 and UM2 fractions and M and UDP3 fractions in proportions greater than those with rAly1.

**FIG 8:**
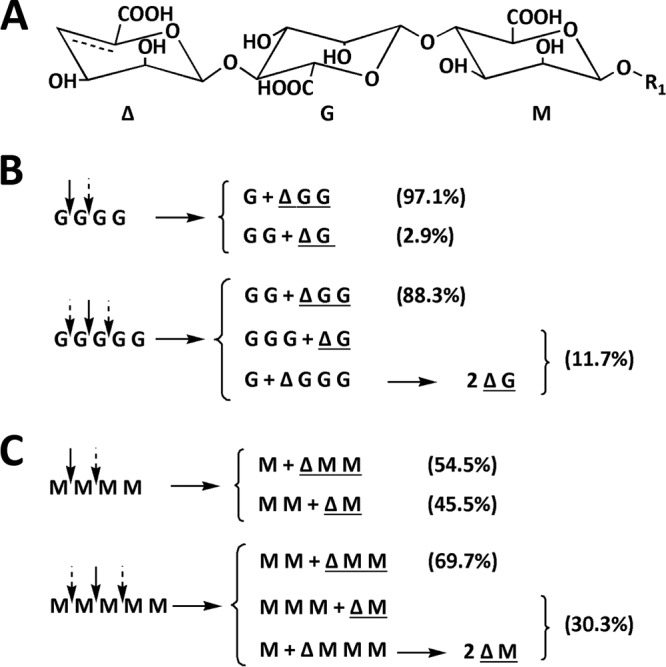
Variable action modes of rAly1 toward saturated alginate oligosaccharide fractions. (A) Structural properties of unsaturated oligosaccharide chains yielded by alginate lyase via a β-elimination mechanism. Δ, unsaturated sugar unit; M, β-d-mannuronate; G, α-l-guluronate. (B and C) Arrows indicate possible cleavage sites of rAly1 toward saturated oligosaccharide chains enriched with G (B) or M (C). Molar proportions of possible oligosaccharide metabolic pathways with rAly1 were calculated by area integration of the underlined product fractions in gel filtration HPLC results.

To compare the action modes of rAly1 and rAly1-T185N toward guluronate and mannuronate, various size-defined saturated oligosaccharide fractions were individually reacted with the two enzymes, using the same procedure described for unsaturated saccharide. The results (Fig. 7A) showed that rAly1 could not degrade G2 and G3 but could digest larger size-defined fractions, e.g., G4 and G5, individually, yielding unsaturated G-enriched disaccharide (UG2) and trisaccharide (UG3) fractions as the final main unsaturated products (Fig. 7A). As previously demonstrated in this paper (Fig. 6A), the enzyme rAly1 cannot degrade UDP4 fractions to produce Δ and UDP3 products or digest UDP3 fractions to produce Δ and UDP2 products; however, it could only partially degrade UDP4 fractions to produce UDP2 products. Thus, rAly1 could digest G4 to yield the monosaccharide G and UG3 as the final main products, yielding G2 and UG2 products in a low proportion (∼2.9%) (Fig. 7A and 8B). Moreover, it could degrade G5 to produce G2 and UG3 as the final main products, yielding a low proportion (∼11.7%) of G3 and UG2 products or few G and UG4 products (Fig. 7A and 8B). The results demonstrated that G4 is the smallest saturated G-enriched oligosaccharide substrate and that G is the minimal saturated G-enriched oligosaccharide product of Aly1. Furthermore, the bifunctional alginate lyase Aly1 produces various size-defined saturated oligosaccharide products, i.e., G, G2, and G3, from the NR ends of G4 or larger saturated G-enriched substrates, with size enlargement of the main products parallel to the corresponding substrates (Fig. 8B).

When reacted with saturated G-enriched oligosaccharides, the NCR-truncated enzyme rAly1-T185N showed alginate-degrading properties similar to those of rAly1 (Fig. 7A), indicating that deletion of the NCR fragment of Aly1 led to rare degradable size changes or degradation efficiency changes toward saturated G-enriched oligosaccharide substrates.

Similar to the degradation of saturated G-enriched oligosaccharides, rAly1 could not degrade M2 and M3 chains but could digest larger size-defined fractions, e.g., M4 and M5, to yield unsaturated M-enriched disaccharide (UM2) and trisaccharide (UM3) fractions as the final unsaturated products (Fig. 7B). Furthermore, rAly1 could degrade M4 to produce M and UM3 or M2 and UM2 fractions as the final main products, with the yield of UM3 to UM2 at a molar ratio of ∼1.2:1 (Fig. 7B). The enzyme could digest M5 to produce M2 and UM3, or M3 and UM2, as the final products, with a UM3-to-UM2 molar ratio of ∼2.3:1 (Fig. 7B). Weak signals for M and UM4 products were detected by time course analysis (data not shown). These results demonstrated that M4 is the smallest M-enriched saturated oligosaccharide substrate and that M is the minimal saturated oligosaccharide product of Aly1. Furthermore, the bifunctional alginate lyase Aly1 can yield various size-defined saturated M-enriched oligosaccharide products, i.e., M, M2, and M3 from the NR ends of M4 or larger size-defined M-enriched oligosaccharide chains, with parallel size enlargements of the main product fractions to the corresponding substrates (Fig. 8C).

When reacted with saturated M-enriched oligosaccharide samples, the NCR-truncated protein rAly1-T185N showed degradation patterns similar to those of rAly1 but could digest M4 and M5 at proportions greater than those for rAly1 to produce more unsaturated products of the UM2 and UM3 fractions (Fig. 7B and 8C). Moreover, it could degrade M5 to produce more UM3 than UM2 products, with a molar proportion of ∼2.4:1 (Fig. 7B). The results indicated that via NCR truncation, the mutant protein increased the yields of M, M2, and M3 from the NR ends of M4 and larger size-defined saturated M-enriched substrates, along with parallel size enlargements of the main product fractions to the corresponding substrates (Fig. 7B and 8C). Furthermore, deletion of the NCR fragment of Aly1 increased the truncated protein's enzyme activity toward small size-defined saturated M-enriched oligosaccharide substrates (Fig. 8C), thereby enhancing the M preference and alginate-degrading efficiency.

### Degradation pattern for fluorescently labeled oligosaccharides.

To investigate the variable alginate-degrading model of Aly1, various saturated oligosaccharide fractions enriched with M or G were individually labeled using excess 2-aminobenzamide (2-AB). The resulting products were purified via gel filtration and reacted with rAly1 or rAly1-T185N.

**FIG 9:**
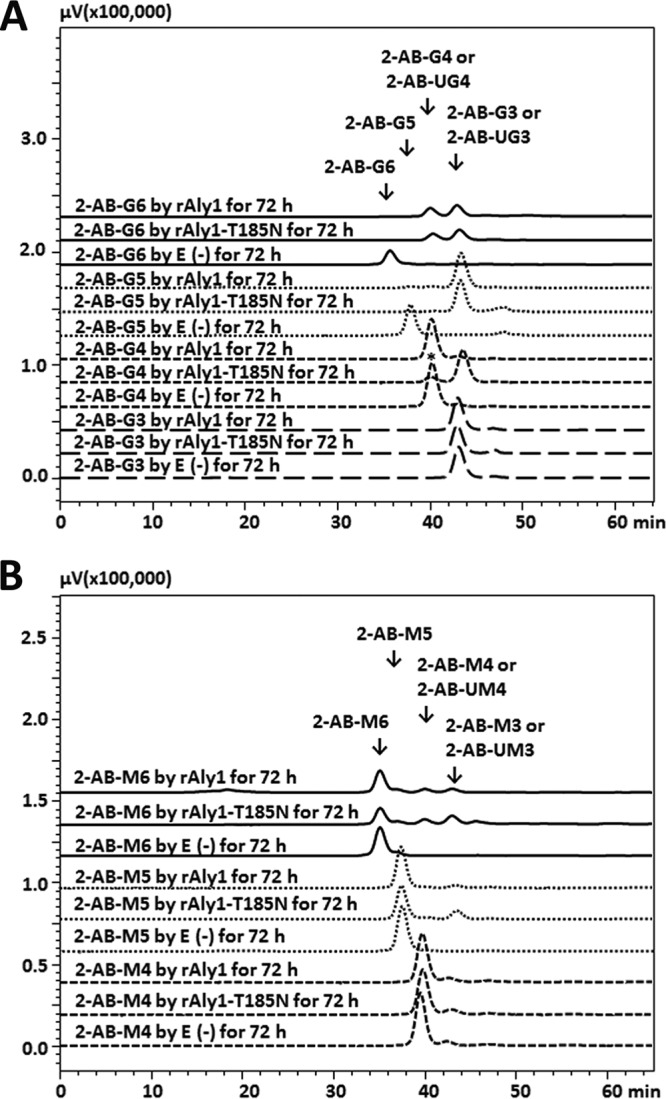
Fluorescent HPLC analyses of final digests of 2-AB-labeled saturated G-enriched fractions (A) or M-enriched fractions (B) produced by rAly1 and NCR-truncated protein rAly1-T185N. E(-), without the enzyme. Fluorescent HPLC analyses were performed using a Superdex peptide 10/300 GL column with an excitation wavelength of 330 nm and a monitoring wavelength of 420 nm. An asterisk indicates that the enzyme rAly1-T185N degraded 2-AB-G4, 2-AB-M5, and 2-AB-M6 in proportions greater than those with rAly1.

The enzyme rAly1-T185N, as well as rAly1, did not digest 2-AB-labeled oligosaccharides of G3 or UG3 (Fig. 9A). The enzyme rAly1 could only slightly degrade 2-AB-labeled G4 fractions, whereas rAly1-T185N could almost completely digest 2-AB-labeled G4 chains to produce G and 2-AB-labeled UG3 products (Fig. 9A). Similarly, the two enzymes could completely digest the 2-AB-labeled G5 fraction to produce G2 and 2-AB-labeled UG3 fractions as the final main products (Fig. 9A), but they yielded few G and 2-AB-labeled UG4 products in the time course analysis (data not shown). These results indicated that deletion of the NCR fragment of Aly1 leads to enhancement of the efficiency of degradation of 2-AB-labeled G4 fractions, i.e., increased G-producing capabilities, by the truncated protein.

When reacted with 2-AB-labeled saturated M-enriched oligosaccharides, similar to the case for rAly1, the NCR-truncated enzyme rAly1-T185N did not digest 2-AB-labeled oligosaccharide fractions of M3, UM3, or M4 (Fig. 9B). The enzyme rAly1 degraded 2-AB-labeled M5 in a low proportion (2%) to produce M2 and 2-AB-labeled UM3, whereas rAly1-T185N digested 2-AB-labeled M5 in a greater proportion of 4.2% to produce M2 and 2-AB-labeled UM3 as products (Fig. 9B). These results indicated that deletion of the NCR fragment of Aly1 leads to weakly enhanced efficiency of degradation of 2-AB-labeled M5, i.e., a weak increase in M2-producing capabilities of the truncated protein.

## DISCUSSION

Bacterial strains of the genus Flammeovirga showed excellent capabilities of growing on alginate as the sole carbon source (29–32), suggesting that they are resources for variously efficient alginate-degrading enzymes. To date, only a G-specific endolytic alginate lyase, Aly5, has been identified from Flammeovirga sp. strain MY04 (27). In this study, although the protein Aly1 encoded by the MY04 genome shared only slight homology with the elucidated enzymes, it has been predicted to be a novel member of the PL7 superfamily because it contains the conservative motif Gln^318^-Ile^319^-His^329^ in the hypothetical catalytic module Alg2 (see Fig. S1 in the supplemental material). The recombinant enzyme rAly1 showed high enzyme activities, in reducing order, for alginate, G blocks, and M blocks (Table 1), which is a primary characteristic of a bifunctional alginate lyase with G preference. However, convincing data are still lacking. Remarkably, small size-defined unsaturated oligosaccharide fractions obtained from the final alginate digests of Aly1 showed a successional rule in their structural properties (Fig. 5; see Fig. S4 in the supplemental material); i.e., the UDP2 fractions contain only ΔG units, and the UDP3 fractions contain the NR ends of ΔG as well as ΔM units, whereas larger products, including UDP4 fractions, contain only ΔM ends, similar to the oligosaccharide-yielding property described for the G-specific endolytic alginate lyase Aly5 of strain MY04 (27). Moreover, when reacted with saturated G-enriched and saturated M-enriched oligosaccharide fractions under comparable conditions, the enzyme rAly1 yielded unsaturated G-enriched oligosaccharide products with a specific *A*_235_ that was 6- to 10-fold higher than that of M-enriched ones (Fig. 7A and B); i.e., Aly1 degraded G-enriched sugar chains at higher proportions than M-enriched fractions. Thus, Aly1 is demonstrated to be a bifunctional alginate lyase that prefers G to M.

In addition to the broad substrate spectrum, the bifunctional endolytic alginate lyase Aly1 exhibits more enzymatic properties that are suitable for efficient alginate degradation than the G-specific endolytic alginate lyase Aly5 (27); e.g., the enzyme activity of Aly1 to alginate is almost 3-fold that of Aly5 at an equimolar amount of enzyme, the smallest substrates of Aly1 (the M4, G4, and UDP4 fractions) are smaller than those of Aly5 (the G5 and UDP5 fractions), and the final main alginate degradation products of Aly1 (the UDP2 and UDP3 fractions) are smaller than those of Aly5 (the UDP3 and UDP4 fractions). Notably, monosaccharide products of M and G (Fig. 7A and B and 8B and C), which can support bacterial growth or direct biomass bioconversion, have been found in the final digests of saturated oligosaccharide substrates (e.g., the M4, G4, M5, and G5 fractions) produced by rAly1 but not rAly5. Therefore, the bifunctional endolytic alginate lyase Aly1 degrades alginate more efficiently than the G-specific endolytic alginate lyase Aly5, primarily due to the capability of degrading M-enriched sugar chains and smaller size-defined oligosaccharide substrates.

NCR truncation of module-organized polysaccharide depolymerases, e.g., agarase (38) and alginate lyase (27, 28), commonly causes negative changes in the enzymatic properties of the truncated enzymes, such as weaker stabilities and lower enzyme activities. Moreover, NCR truncation of the bifunctional alginate lyase Aly2 from Agarivorans sp. strain L11 altered substrate preference (28). Similar protein truncation of the G-specific alginate lyase Aly5 from Flammeovirga sp. strain MY04 has led to rare substrate preference changes but size enlargement of the degradable oligosaccharide fractions (27). These previous studies have provided various beneficial methods for enzyme improvement of wild-type alginate lyases. However, relatively few have shown positive results, and the corresponding mechanisms are poorly understood. In this study, NCR truncation of Aly1 showed weak effects on the truncated enzyme's biochemical characteristics but increased enzyme activity toward alginate to 1.34-fold at an equimolar amount of enzyme (Table 1), which is a positive result and different from that for the NCR truncation of the bifunctional alginate lyase Aly2 or NCR truncation of the guluronate lyase Aly5. Furthermore, although the NCR-truncated enzyme rAly1-T185N degraded alginate in patterns similar to those of rAly1 and produced final oligosaccharide products with similar characteristics, e.g., molecular sizes, molar ratios, and structural properties (Fig. 4A, 5, and S4), it enhanced the degradation proportion of small size-defined saturated M-enriched oligosaccharide substrates (e.g., the M4 and M5 fractions) (Fig. 7B) and unsaturated substrates, including UDP4 fractions (Fig. 6B). It showed no degradation pattern changes for saturated G-enriched oligosaccharide chains (Fig. 7A) or size changes of the associated degradable oligosaccharide fractions. Thus, we have provided possible mechanisms by which NCR truncation of the bifunctional endolytic alginate lyase Aly1 enhances M preference and improves alginate degradation efficiency, which will be beneficial for improvement of associated enzymes.

Recombinant proteins of Aly1 and its catalytic module Alg2 exhibited distinct substrate-degrading patterns and oligosaccharide-yielding properties in the degradation of alginate and various size-defined oligosaccharide substrates, including saturated, unsaturated, and 2-AB-labeled saturated types. Our study demonstrated that a bifunctional endolytic alginate lyase degrades small size-defined oligosaccharide substrates using variable substrate-degrading modes (Fig. 8B and C). Furthermore, the terminus types, molecular sizes, and M/G contents of oligosaccharide substrates are key factors that can cause mode changes of the enzymes. Comparisons and the resulting novel mechanisms are described as follows.

First, when digesting saturated G4 or M4 fractions, the two enzymes yielded various small size-defined products of saturated oligosaccharides (i.e., the M, G, M2, and G2 fractions) and unsaturated oligosaccharides (i.e., the UDP2 and UDP3 fractions), with monosaccharide units of M and G as the final main saturated products (Fig. 7A and B). While degrading UDP4 fractions, they produced only UDP2 products (Fig. 6A and B). Therefore, saturated units of M or G, rather than the unsaturated unit Δ, at the nonreducing ends of sugar chains are essential for the catalytic module Alg2 of Aly1 to act in monosaccharide-producing modes, i.e., to degrade associated substrates completely and efficiently. In contrast, the unit Δ is essential for the enzymes to cleave oligosaccharide products ≥UDP2 from substrates. Second, when digesting larger size-defined saturated oligosaccharide substrates, e.g., M5 or G5 fractions, the two enzymes produced larger sugar chains, i.e., M2 and M3 or G2 and G3 fractions, as the final main saturated oligosaccharide products (Fig. 7A and B), indicating parallel size enlargement of saturated products to saturated substrates. Similar mode changes were observed when we compared the yields of unsaturated oligosaccharide products from the UDP4, UDP5, and UDP6 fractions (Fig. 6A and B) by the two enzymes. Thus, the catalytic module Alg2 of Aly1 can vary the size of its catalytic tunnel to produce various size-defined oligosaccharide products in accordance with the molecular sizes of the oligosaccharide substrates. Third, the two enzymes produced G and UG3 fractions as the final main digests of G4 fractions, while they yielded approximately equal proportions of M and UM3 and of M2 and UM2 fractions as the final main products of the M4 fractions (Fig. 7A and B). Similar product differences were also observed when we compared the degradation patterns of G5 and M5 fractions (Fig. 7A and B). Therefore, the M/G contents of oligosaccharide substrates have strong effects on the oligosaccharide-yielding properties of the enzymes; this may be because the catalytic module Alg2 of Aly1 is bifunctional and prefers G to M.

Notably, the G4, M4, and UDP4 fractions are the smallest natural substrate types of rAly1 and rAly1-T185N (Fig. 6A and B and 7A and B). However, 2-AB-labeled G5 and 2-AB-labeled M5 fractions are the smallest artificially synthesized substrate types of rAly1, and 2-AB-labeled G5 and 2-AB-labeled M4 fractions are the smallest artificial substrate types of rAly1-T185N (Fig. 9A and B). Therefore, 2-AB labeling at the reducing (R) ends of oligosaccharide chains can cause a stereospecific blockade that enlarges the sizes of degradable oligosaccharide fractions. Thus, the enzyme rAly1 could no longer produce any monosaccharide products of M or G from 2-AB-labeled oligosaccharide chains (Fig. 9A). Similarly, rAly1-T185N lost its M-producing capability (Fig. 9B) but retained the ability to yield G units by almost completely degrading 2-AB-labeled G4 chains (Fig. 9A). Moreover, rAly1-T185N could degrade 2-AB-labeled G5 and 2-AB-labeled M5 fractions at greater proportions than rAly1 (Fig. 9A), similar to the degradation of G5 versus M5 fractions (Fig. 7A and B). However, the two enzymes produced only saturated disaccharide fractions (M2 or G2) without any saturated trisaccharide products (G3 or M3) from the NR ends of 2-AB-labeled saturated pentasaccharide fractions (Fig. 9A and B), which is notably different from the degradation patterns of M5 and G5 fractions (Fig. 7A and B). Thus, 2-AB labeling at the R end can prevent Aly1 and its catalytic module Alg2 from cleaving various size-defined saturated oligosaccharide product fractions from the NR ends of single sugar chains. It can retain the capability of producing various size-defined oligosaccharide products from different size-defined sugar chains and thus inhibit action mode changes of enzymes.

### Conclusions.

In addition to the previously reported G-specific endolytic alginate lyase Aly5, the protein Aly1 of Flammeovirga sp. strain MY04 is a novel member of the PL7 superfamily. It is bifunctional and prefers G to M. Notably, the catalytic module of Aly1 can vary its endolytic modes in accordance with terminus types, molecular sizes, and M/G contents of substrates, yielding various small size-defined saturated oligosaccharide products (including monosaccharide) and unsaturated oligosaccharide products (≥UDP2) from single or different saturated sugar chains with a parallel size enlargement of products to substrates. NCR truncation of Aly1 can enhance the degradation proportions of small size-defined saturated M-enriched oligosaccharide substrates and intermediate products, including UDP4 fractions, thus enhancing the M preference and enzymatic activity. The 2-AB labeling of oligosaccharide substrates can cause size enlargement of degradable oligosaccharide fractions and inhibit the yields of various size-defined saturated oligosaccharide products from single, but not different, sugar chains.

## MATERIALS AND METHODS

### Bacterial strains, carbohydrates, and growth conditions.

Agarose, alginate (viscosity, ≥2,000 cP; 2% [25°C]), chondroitin, chondroitin sulfates (A, C, and E types), dermantant sulfate (B type), hyaluronan, heparin, heparin sulfates, and xanthan were purchased from Sigma-Aldrich Co. Ltd., USA. G blocks, M blocks, and standard size-defined M-enriched or G-enriched saturated sugar chains (i.e., disaccharide, trisaccharide, tetrasaccharide, and pentasaccharide, with >95% promised purities) were purchased from Qingdao BZ Oligo Biotech Co. Ltd. (Qingdao, China), and various size-defined unsaturated oligosaccharide fractions were prepared from partial alginate digests with the alginate lyase Aly5 (27).

Flammeovirga sp. strain MY04 (CGMCC no. 2777) was cultured at 30°C in a medium (pH 7.0) containing (wt/vol) 0.40% tryptone, 0.25% yeast extract, and 3.0% NaCl (32). Escherichia coli strains were cultured at 37°C in Luria-Bertani (LB) broth supplemented with ampicillin (100 μg/ml) or kanamycin (50 μg/ml) when necessary. Agar powder (1.5%, wt/vol) was used to prepare the solid media.

### Sequence analyses of genes and proteins.

Using BioEdit version 7.2.5 (39), the DNA sequence of ORF2539 was translated into the amino acid sequence of the Aly1 protein, and the GC content (%G+C) of the open reading frame (ORF) was calculated. An online similarity search of the protein sequence was performed using the BLAST algorithm on the National Center for Biotechnology Information server (http://www.ncbi.nlm.nih.gov). The signal peptide was identified using the SignalP 4.1 server (http://www.cbs.dtu.dk/services/). Molecular weights of the proteins were estimated using the peptide mass tool on the ExPASy server of the Swiss Institute of Bioinformatics (http://swissmodel.expasy.org/). Protein modules and domains were identified using the Simple Modular Architecture Research Tool (https://en.wikipedia.org/wiki/Simple_Modular_Architecture_Research_Tool), the Pfam database (http://pfam.xfam.org), and the Carbohydrate-Active Enzyme database (http://www.cazy.org). Multiple-sequence alignments and phylogenetic analyses were performed using MEGA version 6.01 (40).

### Construction of expression vectors.

Genomic DNA from Flammeovirga sp. strain MY04 was prepared and purified using the TIANamp bacterial DNA kit (Tiangen Biotech Co. Ltd., Beijing, China). To express the whole protein, the full-length gene of Aly1 was amplified from genomic DNA using high-fidelity PrimeSTAR HS DNA polymerase (TaKaRa, Dalian, China) and the primers E30L-1f (5′-GGATCCAACAATAAAGTAGAGGACGAG-3′) and E30L-1r (5′-CTCGAGTATAAGTTTCTTTTAATTCTATAG-3′). Primer pairs with restriction enzyme sites (underlined) for NdeI and XhoI were designed to generate a His_6_ tag at the C terminus of the recombinant protein rAly1. PCR products were gel recovered and enzyme cloned into the expression vector pET-30a(+), yielding the recombinant plasmid pET30-Aly1. To express an NCR-truncated protein of His_6_-tagged rAly1, i.e., the recombinant protein of the putative catalytic module Alg2 (rAly1-T185N), the pET30-Aly1 plasmid was amplified using the high-fidelity DNA polymerase in the PrimeSTAR Max premix (TaKaRa, Dalian, China) and the primers T185N-F (5′-TACAAAGAAGACGAAGTGCCAG-3′) and T185N-R (5′-GGATCCGATATCAGCCATGGC-3′). The PCR products were gel recovered, terminus phosphorylated, and circle ligated to yield the plasmid pET30-Aly1-T185N. The integrities of the nucleotide sequences of newly constructed plasmids were confirmed by DNA sequencing.

### Heterologous expression and purification of rAly1 and rAly1-T185N.

rAly1 and rAly1-T185N were expressed and purified using the same protocol described for the recombinant alginate lyase rAly5 of Flammeovirga sp. strain MY04 (27). Briefly, the plasmids pET30-Aly1 and pET30-Aly1-T185N were individually transformed into E. coli BL21(DE3) cells. To initiate gene expression, LB broth was supplemented with isopropyl-1-thio-β-d-galactoside to a final concentration of 0.05 mM when the cell density reached an *A*_600_ value of 0.6 to 0.8. After 24 h of continual cultivation at 16°C, cells were harvested by centrifugation at 6,000 × *g* for 10 min, washed twice with ice-cold buffer A (50 mM Tris, 150 mM NaCl, pH 8.0), resuspended in buffer A, and disrupted by sonication (60 repetitions, 5 s). After centrifugation at 15,000 × *g* for 30 min, the supernatant containing each soluble protein was loaded onto a buffer A-equilibrated Ni-nitrilotriacetic acid agarose (Ni-NTA) column (Novagen, USA). Subsequently, each column was eluted with buffer containing imidazole in gradient concentrations, i.e., 0, 10, 50, and 250 mM. Fractionated samples were analyzed using SDS-PAGE. To obtain active alginate lyases, purified protein factions were dialyzed against buffer B (50 mM Tris, 50 mM NaCl, 5% [vol/vol] glycerol, pH 8.0).

SDS-PAGE was performed using 13.2% (wt/vol) polyacrylamide gels according to the methods of Sambrook and Russell (41). Proteins were detected by staining the gels with Coomassie brilliant blue R-250. Protein concentrations were individually determined by the Folin-Lowry method using Folin Ciocalteu's phenol reagent (Sigma-Aldrich, USA) with bovine serum albumin as the standard.

### Enzyme activity assay.

To determine the substrate preferences of rAly1 and rAly1-T185N, various polysaccharides were individually dissolved in deionized water to prepare stock solutions (3 mg/ml). Each stock solution (100 μl) was mixed with 30 μl of appropriately diluted enzyme, 100 μl of 150 mM NaAc-HAc buffer (pH 6.0), and 70 μl of water. Each reaction mixture was incubated at 40°C for 12 h. Enzyme-treated samples were heated in boiling water for 10 min and subsequently ice cooled. After centrifugation at 15,000 × *g* for 15 min, the supernatant was collected and analyzed by measuring the absorbance at 235 nm. One unit was defined as the amount of enzyme required to increase the absorbance at 235 nm by 0.1 per min (27).

### Biochemical characterization of rAly1 and rAly1-T185N.

To determine the optimal temperature for alginate lyase activities, alginate, G blocks, and M blocks were individually reacted with the two protein preparations. Enzymatic reactions were performed in 50 mM NaAc-HAc buffer (pH 6.0) at temperatures ranging from 0 to 80°C for 90 min. After the optimal temperature was determined, the effects of pH on enzyme activity were tested in different buffers, including 50 mM NaAc-HAc buffer (pH 4.0 to 6.0), 50 mM NaH_2_PO_4_-Na_2_HPO_4_ buffer (pH 6.0 to 8.0), and 50 mM Tris-HCl buffer (pH 7.5 to 10), each in a total volume of 300 μl. The thermostability was evaluated by measuring the residual enzyme activity of each enzyme after incubation for 0 to 24 h at temperatures ranging from 0 to 80°C. The effects of pH on enzyme stability were determined by measuring the residual activity of each enzyme after incubation at 4°C at various pH values (4.0 to 10) for 2 h. The effects of metal ions and chelating agents on alginate lyase activities were examined by determining the activity of each enzyme in the presence of 1 mM and 10 mM concentrations of various chemicals, respectively. All reactions were performed in triplicate. After each treatment, the enzyme activity was estimated by measuring the absorbance at 235 nm.

### Comparison of the polysaccharide degradation properties of rAly1 and rAly1-T185N.

To compare the polysaccharide degradation patterns of rAly1 and rAly1-T185N, alginate (1.0 mg/ml) digestion by each enzyme (1.0 U/ml) at 40°C was traced over 72 h. Similar experiments were performed at different final alginate concentrations, ranging from 1.0 mg/ml to 10 mg/ml. Aliquots of the digests were removed for time course analysis. To determine the molar ratio of each oligosaccharide fraction in the products, samples (1.0 mg/ml) were analyzed by gel filtration on a Superdex peptide 10/300 GL column (GE Healthcare, USA) and monitored at 235 nm using a UV detector. The mobile phase was 0.2 M NH_4_HCO_3_, and the flow rate was 0.4 ml/min. Online monitoring and data analysis were performed using LCsolution version 1.25 software.

To determine the oligosaccharide compositions of the final digests, 100 mg alginate (1.0 mg/ml) was digested by excess enzyme (10 U/ml each) at 40°C for 72 h. To obtain size-defined unsaturated oligosaccharide fractions, the final alginate degradation products of each enzyme were gel filtered through a Superdex peptide 10/300 GL column using the same protocol described above. Each fraction was collected and freeze-dried repeatedly to remove NH_4_HCO_3_ for further analysis. The molecular mass of each oligosaccharide fraction was determined by matrix-assisted laser desorption ionization–time of flight mass spectrometry (AXIMA-CFR plus; Shimadzu, Japan). For ^1^H-NMR spectroscopy, each purified oligosaccharide fraction (2 mg) was dissolved in 0.3 ml of D_2_O in 5-mm NMR tubes. The spectra were recorded on a JNM-ECP600 (JEOL, Japan) apparatus set at 600 MHz, using tetramethylsilane (TMS) as the internal standard.

### Comparison of oligosaccharide degradation properties of rAly1 and rAly1-T185N.

To determine the smallest substrate for each enzyme, unsaturated oligosaccharides with differing degrees of polymerization (DPs), i.e., the UDP2, UDP3, UDP4, UDP5, UDP6, and UDP7 fractions, were reacted with rAly1 and rAly1-T185N using the same strategy as described previously for rAly5 (27). To determine the substrate preferences of the two enzymes, standard size-defined M-enriched and G-enriched saturated sugar chains were individually reacted with the two enzymes. The above-mentioned natural substrates and their enzymatic products (20 μg each) were subjected to the gel filtration assay described above and monitored at 235 nm using a UV detector.

To compare the enzymatic degradation patterns of the two enzymes, various size-defined saturated and unsaturated oligosaccharide fractions were fluorescently labeled at their reducing ends using excess 2-AB (Sigma-Aldrich, USA), as described by Bigge et al. (42). The labeled products (∼1 μg each) were purified by gel filtration HPLC and further degraded with each enzyme (5 U) in a total volume of 1 ml using the protocol described above. The above-mentioned artificial substrates and their enzymatic products (50 ng each) were subjected to the gel filtration assay described above using a fluorescence detector and excitation and emission wavelengths of 330 and 420 nm, respectively.

### Accession number(s).

The protein sequence of Aly1 has been deposited at GenBank under accession number ANQ49908.1.

## Supplementary Material

Supplemental material
