# Supplementary material for: Biochemical Characteristics and Variable Alginate-Degrading Modes of a Novel Bifunctional Endolytic Alginate Lyase
Source: Appl Environ Microbiol. 2017 Nov 16;83(23):e01608-17. doi: 10.1128/AEM.01608-17 (PMC5691422; doi:10.1128/AEM.01608-17)
Supplement: Supplemental material [file supp_83_23_e01608-17__index.html]

Supplemental material 

# Biochemical Characteristics and Variable Alginate-Degrading Modes of a Novel Bifunctional Endolytic Alginate Lyase

## Supplemental material

- Supplemental file 1 -

  Protein sequence alignment of Aly1 and characterized PL7 alginate lyases (Fig. S1); nature PAGE analysis (Fig. S2); biochemical characteristics of the NCR-truncated protein rAly1-T185N (Fig. S3); 1H-NMR analysis (Fig. S4).

  PDF, 1.3M
