# Supplementary material for: Biochemical Characteristics and Variable Alginate-Degrading Modes of a Novel Bifunctional Endolytic Alginate Lyase
Source: Appl Environ Microbiol. 2017 Nov 16;83(23):e01608-17. doi: 10.1128/AEM.01608-17 (PMC5691422; doi:10.1128/AEM.01608-17)
Supplement: Supplemental material [file AEM.01608-17_zam023178157s1.pdf]

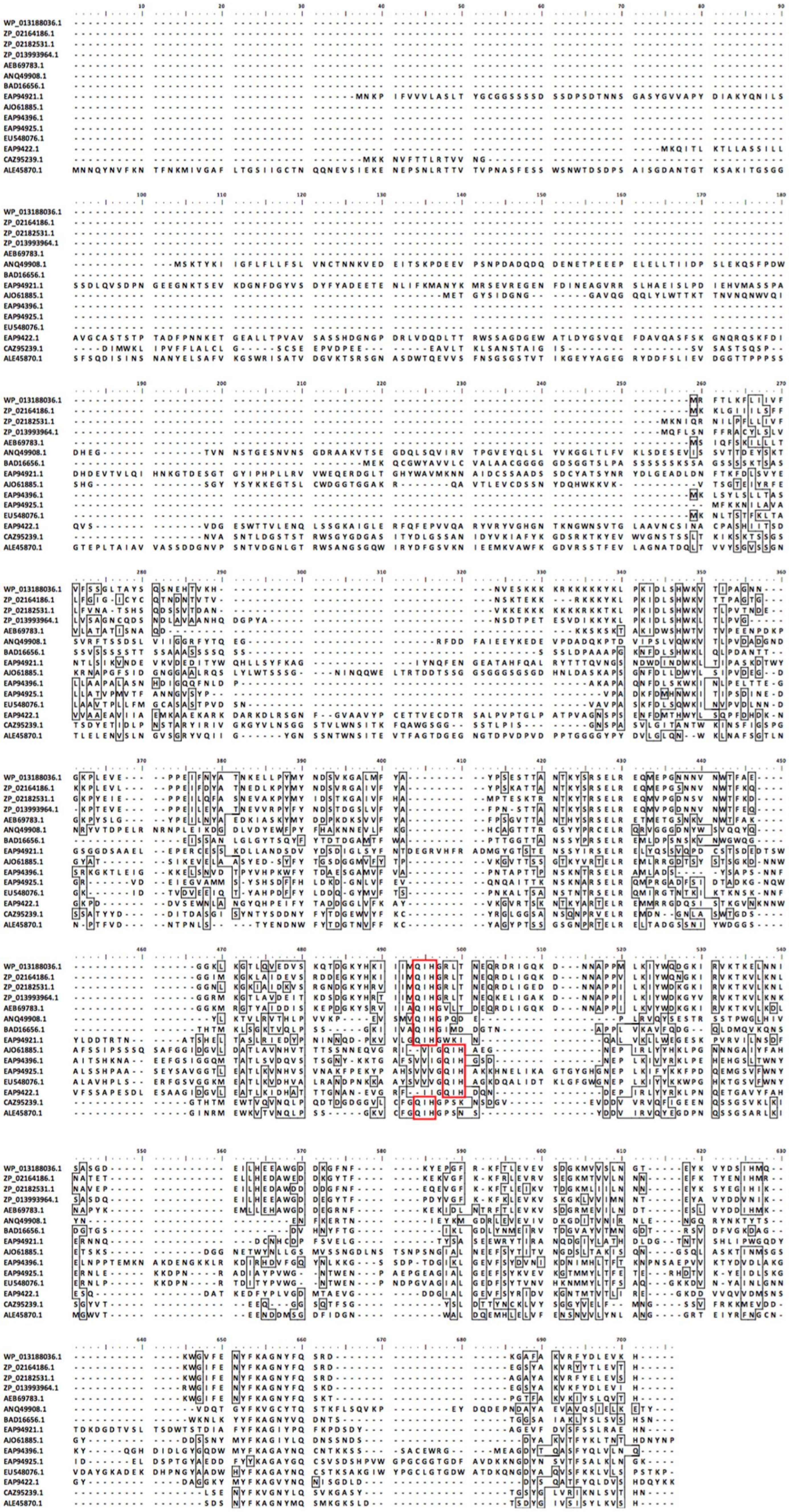
WP_013188036.1
ZP_02164186.1
ZP_02182531.1
ZP_013993964.1
AEB69783.1
ANQ49908.1
BAD16656.1
EAP94921.1
AJO61885.1
EAP94396.1
EAP94925.1
EU548076.1
EAP9422.1
CAZ95239.1
ALE45870.1

FIG S1 Protein sequence alignment of Aly1 and characterized PL7 alginate lyases. Amino acid residues with homology ≥50% are shaded in black frames. The conserved QIH motif in aligned alginate lyases is indicated in red. Alginate lyases and their resources are WP 013188036.1, AlyPG of *Croceibacter atlanticus* strain HTCC2559; ZP 02164186.1, *Kordia algicida* strain OT-1; ZP 02182531.1, *Flavobacteriales bacterium* strain ALC-1; ZP 013993964.1, endo-guluronate lyase AlyA1 of *Zobellia galactanivorans*; AEB69783.1, alginate lyase Alg2A of *Flavobacterium* sp. strain S20; ANQ49908.1, bifunctional alginate lyase Aly1 of *Flammeovirga* sp. strain MY04; BAD16656.1, alginate elyase AlyA1-II' of *Sphingomonas* sp. strain A1; EAP9422.1, alginate lyase AlyB of *Vibrio splendidus* strain 12B01; AJO61885.1, alginate lyase AlyL2 of *Agarivorans* sp. strain L11; EAP94396.1, alginate lyase AlyE of *Vibrio splendidus* strain 12B01; EAP94925.1, alginate lyase AlyD of *Vibrio splendidus* strain 12B01; EU548076.1, alginate lyase AlyPM of *Pseudoalteromonas* sp. strain SM0524; EAP94921.1, alginate lyase AlyA of *Vibrio splendidus* strain 12B01; CAZ95239.1, alginate lyase AlyA1 of *Zobellia galactanivorans*; ALE45870.1, and endo-guluronate lyase Aly5 of *Flammeovirga* sp. strain MY04.

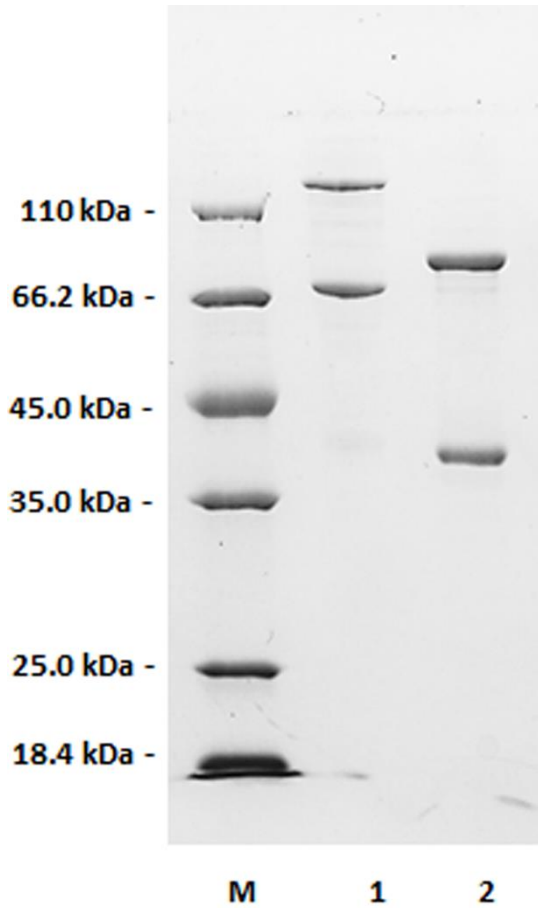

FIG S2 Nature PAGE analysis (13.2%, w/v) of the dimer form of rAly1 and NCR-truncated protein rAly1-T185N. M, standard protein molecular weight marker; 1, rAly1 and its dimer; 2, rAly1-T185N and its dimer.

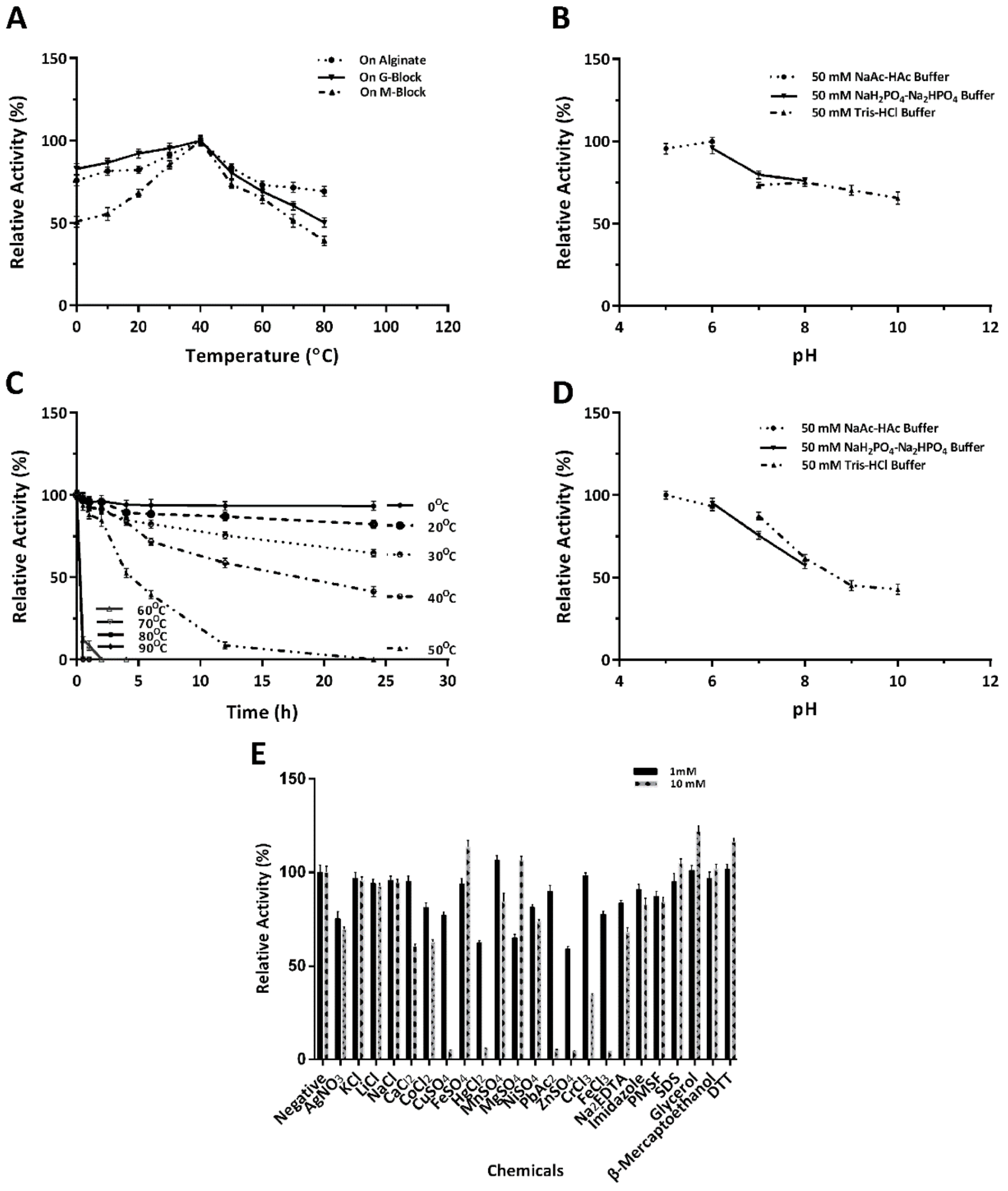

FIG S3 Biochemical characteristics of the NCR-truncated protein rAly1-T185N. A, effects of temperature on enzyme activity towards alginate, G-block, and M-block. B, effects of pH values on enzyme activity towards alginate. C, thermostability of rAly1-T185N towards alginate. D, pH stability of rAly1-T185N towards alginate. E, effects of chemicals on enzyme activity towards alginate.

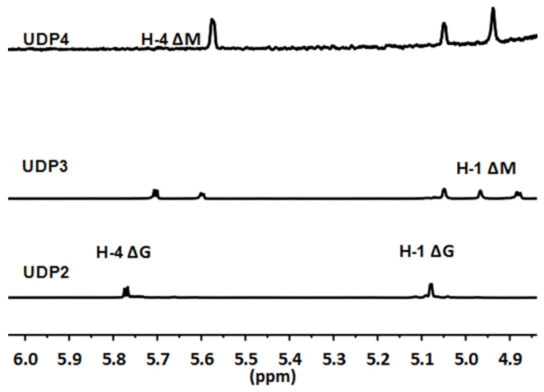

FIG S4 ^{1}H-NMR analysis (600 MHz, 28°C) of various size-defined final oligosaccharide product fractions produced by rAly1-T185N. The H-4Δ signals at 5.70 or 5.74 ppm indicate that ΔG constitutes the first two sugar residues at the nr ends. The H-4Δ signal at 5.54 or 5.57 ppm indicates that ΔM constitutes the first two residues at the nr ends.
